# Effect of Low-dose Ketamine on Inflammatory Markers, Perioperative Analgesia, and Chronic Pain in Patients Undergoing Laparoscopic Inguinal Hernia Surgery: A Prospective, Randomized, Double-blind, Comparative Study

**DOI:** 10.4274/TJAR.2024.241771

**Published:** 2024-12-16

**Authors:** Shivani Vijayakumar Hallikeri, Renu Sinha, Bikas Ranjan Ray, Ravindra Kumar Pandey, Vanlal Darlong, Jyotsna Punj, Virinder Kumar Bansal, Renu Saxena

**Affiliations:** 1 All India Institute of Medical Sciences Department of Anaesthesiology, Pain Medicine and Critical Care, New Delhi, India

**Keywords:** Chronic pain, inflammatory markers, inguinal hernia, ketamine, neutrophil lymphocyte ratio, platelet lymphocyte ratio

## Abstract

**Objective:**

The neutrophil lymphocyte ratio (NLR) and platelet lymphocyte ratio (PLR) are indicators of postoperative inflammatory response. Low-dose ketamine has analgesic and anti-inflammatory properties. Inguinal hernia surgery is associated with a higher incidence of chronic pain.

**Methods:**

Sixty patients aged 18-60 years; American Society of Anesthesiologists status I and II who were scheduled for laparoscopic inguinal hernia surgery were included. After the induction of general anaesthesia, a ketamine 0.5 mg kg-1 bolus, followed by a 0.2 mg kg^-1^ h^-1^ infusion (group K) or saline bolus and infusion (group S) was administered until the end of the surgery. Blood samples were collected at various time intervals. Fentanyl requirement, hemodynamics, verbal analog scale (VAS), emergence delirium, recovery, postoperative nausea and vomiting, and chronic pain were recorded.

**Results:**

Median (interquartile range) NLR was 4.63 times increased at 2 hours postoperatively from the baseline in group S [2.07 (1.72-2.79) to 7.91 (5.74-14.7)] as compared to 2.53 times increase in group K [1.85 (1.4-2.61) to 5.45 (2.89-7.61)] (*P*=0.02). The increase in median PLR from baseline to 2 hours postoperatively was greater in group S (2.98 times) than in group K (1.94 times) (*P*=0.02). The NLR and PLR were comparable on POD1 between the groups. Fentanyl requirement was significantly higher in group S compared to Group K both intraoperatively, (*P*=0.01) and two hours postoperatively (*P*=0.047). More patients had chronic pain and VAS scores in group S than in group K (13 vs 5, *P*=0.05).

**Conclusion:**

Low-dose ketamine reduces postoperative inflammatory response, decreases perioperative opioid requirement, and reduces incidence of chronic pain after laparoscopic inguinal hernia surgery with no significant side effects.

**Main Points**

• Low-dose intravenous ketamine bolus followed by infusion reduces inflammatory response, as observed by a significantly lower increase in neutrophil lymphocyte ratio and platelet lymphocyte ratio values at 2 hours postoperatively from baseline in laparoscopic inguinal hernia surgery.

• Low-dose intravenous ketamine bolus followed by infusion reduces perioperative and chronic pain at 3 months after laparoscopic hernia surgery, with minimal delay in recovery and no side effects.

## Introduction

Systemic inflammatory response depends on the release of various hormones, cytokines, and acute phase reactants during surgery.^1, 2^ An optimum inflammatory response would enhance the patient’s recovery, but an excessive inflammatory response may adversely affect the recovery and postoperative outcome due to immune depression and increased susceptibility to sepsis.

The inflammatory response is assessed by immune mediators like interleukins (IL), C-reactive protein (CRP), tumor necrosis factor-alpha (TNF-α), and procalcitonin; which require specific tests and are time-consuming. Neutrophil lymphocyte ratio (NLR) and platelet lymphocyte ratio (PLR) are easy to assess inflammatory response after surgery through complete blood counts.^1, 3^ Studies have shown that NLR and PLR can correlate with IL-6, CRP, and  TNF- levels and can predict postoperative surgical complications and cancer prognosis.^4, 5, 6, 7, 8, 9^

Anaesthesia techniques and anaesthetic agents also affect the inflammatory process perioperatively.^1^ Ketamine reduces inflammation after surgery by acting at various levels in the inflammatory process.^10^ Low-dose ketamine also provides good analgesia during and after surgery by antagonizing N- methyl D- aspartate (NMDA) receptors and by centrally desensitizing pain without significant side effects, such as emergence delirium and sedation.^11, 12, 13, 14^ The incidence of chronic post hernia pain syndrome after laparoscopic inguinal hernia surgery ranges from 6-20% and has adverse implications on morbidity, healthcare costs, and quality of life.^15, 16^ Previous studies have suggested that low-dose ketamine decreases the incidence of chronic pain after various surgeries.^17, 18^

Therefore, we hypothesized that low-dose ketamine infusion would result in decreased inflammatory response and chronic pain after laparoscopic inguinal hernia surgery. The primary objective of the study was to evaluate the effect of low-dose ketamine on the inflammatory response in terms of NLR and PLR. The secondary objectives were to evaluate the effect of low-dose ketamine on perioperative pain, opioid consumption, emergence delirium, awakening, postoperative nausea and vomiting, shivering, nystagmus, and chronic pain at 3 months.

## Methods

This prospective, randomized, double-blind study was approved by the Institutional Ethics Committee for Post Graduate Research All India Institute of Medical Sciences, Ansari Nagal, New Delhi for this study (approval no.: IECPG-268/28.06.2018, date: 26.07.2018) and registered with the Clinical Trials Registry, India, (CTRI/2018/08/015320). Written informed consent was obtained from the patients before recruitment.

Sixty patients aged 18-60 years with American Society of Anesthesiologists I and II who were scheduled for laparoscopic inguinal hernia surgery were randomized using computer-generated random numbers into groups K and S. Patients with a history of epilepsy, recurrent hernia, complicated hernia, conversion from laparoscopic to open surgery, inability to understand the scoring system, and refusal to participate were excluded from the study. Allocation was encealled by the closed envelop method, which was opened in the operating room. The study drugs and infusions were prepared by the anesthesiologist; who was not involved in the study.

Pre-anaesthesia examination was performed one day before surgery, and a blood sample was drawn to obtain a complete blood count at baseline. The verbal analog scale (VAS) was explained to all patients to measure postoperative pain. On the day of surgery, baseline vitals [heart rate (HR), non-invasive blood pressure, SpO_2_] were noted before induction of anaesthesia. Anaesthesia induction was performed with fentanyl 2 µg kg^-1^, propofol 2 mg kg^-1^ followed by atracurium 0.5 mg kg^-1^. In group K, ketamine bolus 0.5 mg kg^-1^ followed by ketamine infusion 0.2 mg kg^-1^ h^-1^ was administered until the end of surgery. In group S, patients received a saline bolus and infusion until the end of the surgery. The airway was secured with an appropriately sized cuffed endotracheal tube. Anaesthesia was maintained with O_2_, air (FiO_2_ 0.5), and isoflurane (MAC 0.8-1.2). Vital signs were noted every 5 minutes.

If HR or mean arterial pressure increased by more than 20% from baseline, 1.0 µg kg^-1^ fentanyl was administered. Paracetamol (1 g) and ondansetron (4 mg) were administered 10 minutes before the end of surgery. At the end of the surgery, after regaining spontaneous breathing, neuromuscular blockade was reversed with a combination of 50 µg kg^-1^ neostigmine and 10 µg kg^-1^ glycopyrrolate. The trachea was extubated after regular respiration with adequate tidal volume. The time from stoppage of isoflurane to extubation and time from stoppage of isoflurane to following verbal commands were noted, and the patient was shifted to the post anaesthesia care unit. Postoperative hemodynamic monitoring, time to reach modified Aldrete score of ≥9, VAS score, emergence of delirium by Richmond agitation sedation scoring system (RASS), shivering, nystagmus, and postoperative nausea or vomiting (PONV) were noted at regular intervals.

If the RASS was +2 or +3, no interventions were performed. If the RASS was 3 or 4, midazolam 0.4 µg kg^-1^ was administered. If the PONV score was 3, ondansetron 4 mg was administered. If still PONV was not controlled then metoclopramide 150 µg kg^-1^ was given. If VAS was 4-6 then fentanyl 0.5 µg kg^-1^ and if VAS was ≥7 then fentanyl 1 mg kg^-1^ was administered.

Patients were shifted to the ward after achieving a modified Aldrete score of ≥9 tramadol 50 mg and paracetamol 1 g twice daily were administered as and when required. On POD1, the patient was followed-up in the ward, and the requirement for analgesics was noted. The patients were discharged with instructions for oral paracetamol 500 mg.

Blood samples (two mL venous blood) were collected in EDTA vials at pre-anaesthetic check-up (T0), 2 hours postoperatively (T1), and on POD1 (T2).

The neutrophil count (NC), lymphocyte count (LC), and platelet count (PC) were noted. The NLR was calculated by dividing the absolute neutrophil count by the absolute lymphocyte count (ALC); PLR was calculated by dividing the absolute platelet count by the ALC.

Three months later, patients were interviewed telephonically for chronic pain. Their VAS, analgesic intake, radiating pain on exertion, pain at rest, throbbing pain, discomfort, wound site infection, and any need for a doctor consultation for a condition related to hernia surgery during the last three months were asked and noted.

### Statistical Analysis

There is no previous study on the reference of changes in NLR and PLR after laparoscopic surgery with low-dose ketamine administration, so we included 60 cases as a pilot study.

Categorical data were summarized according to frequency (percentage). Continuous variables were summarized by mean ± standard deviation, 95% confidence interval (CI), and median [interquartile range (IQR)/minimum and maximum] as appropriate. Qualitative data is compared using the chi-square test between the groups. Quantitative data is compared by Student’s t-test, Kruskal-Wallis test, and Mann-Whitney U test between the groups. STATA 14.0 (2015) statistical software was used for analysis. *P *value <0.05 is considered to be significant.

## Results

**Figure 1:**
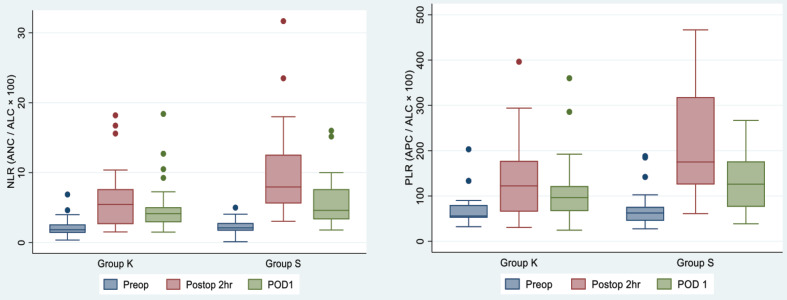
Consort diagram NLR, neutrophil lymphocyte ratio; PLR, platelet lymphocyte rat

Data of 60 patients were included until POD1. At 3 months, telecommunication was not possible in two patients in group K and one patient in group S. Twenty-eight patients in group K and 29 patients in group S were followed up until the end of the study (Figure 1).

Demographic data, type and duration of surgery, and hemodynamic parameters were comparable between both groups (Table 1). The mean intraoperative fentanyl requirement was significantly lower in group K (7±15.2 µg) [95% CI (1.35-12.64 µg)] than in group S (26.6±28.3 µg) [95% CI (16.07-37.2 µg)] (*P*=0.01) (Table 2). The time taken for extubation was comparable between both groups. The time to follow verbal commands (13.7±5.9 vs 10.3±4.0 min) and time taken to reach modified Aldrete’s score >9 (18.6±5.8 vs 16±4.2) were significantly longer in group K than in group S (*P*=0.01 and 0.05 respectively) (Table 2).

Preoperative TLC, PC, NC, LC, NLR, and PLR values were comparable between the groups. TLC and NC increased at T1 and T2 from T0, but the increase in TLC was comparable between the groups. The increase in median (IQR) NC at T1 from T0 values was significantly more in group S [84 (80-88.75)%] in comparison to the group K [77 (67.5-80)%] (p=0.0005) (Table 3). Median (IQR) LC reduction from T0 to T1 was more in group S [29 (24-34.25)% to 11 (6.25-14.5)%] compared to group K [31.5 (25-37.95)% to 14.5 (10.5-21.5)%], which was statistically significant between the groups (*P*=0.01). At T2, NC and LC were comparable between the groups (Table 3). PC decreased at T1 and T2 from T0, but the fall was comparable in both groups.

**Figure 2:**
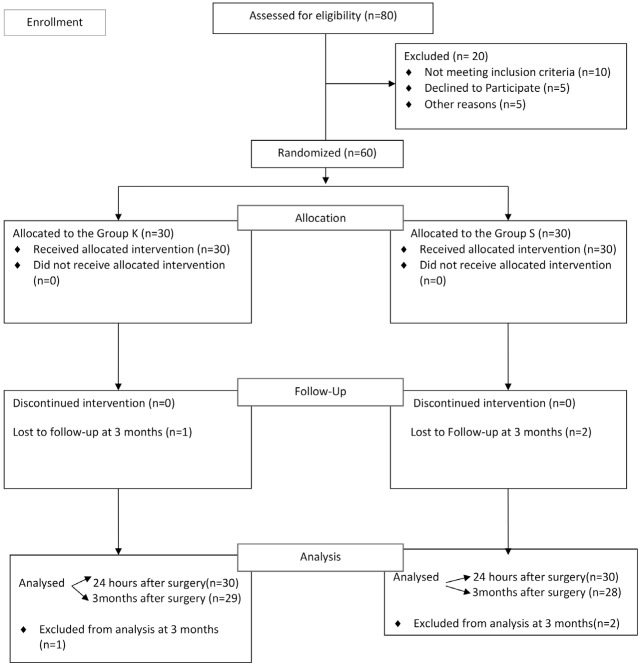
Neutrophil lymphocyte and platelet lymphocyte ratios at different time points Preop, preoperative period; Postop 2hr, postoperative two hours; POD1, postoperative day one; ANC, absolute neutrophil count; ALC, absolute lymphocyte count; APC, absolute platelet count; Mann-Whitney U test was performed, NLR and PLR showed significant rise from preoperative value after two hours of surgery

The NLR at T0 and T2 were comparable in both groups. The NLR showed a statistically significant increase from T0 to T1 in group S compared with group K (*P*=0.007). The median (IQR) NLR in group K was 1.85 (1.4-2.61) at T0, increased to 5.45 (2.89-7.61) at T1, and decreased to 4.14 (2.93-7.61) at T2. In group S, the median (IQR) NLR was 2.07 (1.72-2.79) at T0, which increased to 7.91 (5.74-14.7) at T1 and reduced to 4.21 (3.39-7.55) at T2 (Table 3) (Figure 2).

The PLRs at T0 and T2 were comparable in both groups. The PLR showed a statistically significant increase in group S compared with group K at T1 (*P*=0.03). The median (IQR) PLR at T0 in group K was 55.2 (52.03-78.91) increased to 124.55 (66.45-174.79) at T1 and reduced to 97.77 (66.67-121.41) at T2. In group S, the PLR was 58.9 (45.57-76) at T0, which increased to 157.78 (127.42-305.89) at T1 and reduced to 126.14 (76.31-169.49) at T2 (Table 3) (Figure 2).

Postoperative pain was significantly less in the immediate postoperative phase in group K than in group S [(VAS>3)-3/30 patients vs 13/30 patients] (*P*=0.007). VAS scores at 10, 30, 60, and 120 minutes were comparable in both groups. The total postoperative fentanyl requirement was significantly lower in group K than in group S (40.3±13.7 vs 57±24.4 µg) (p=0.047) (Table 4). The postoperative RASS scores at different time intervals were comparable between both groups. The incidence of PONV, shivering, and nystagmus was minimal in both groups.

At 3 months postoperatively, six patients in group K and 12 patients in group S complained of pain during routine work. Two patients (7.1%) in group K and 11 (37.9%) in group S had a VAS score of 3. One patient (3.6%) in group K and four (13.8%) patients in group S had a VAS score of 4. Significantly more patients in group S had a greater VAS score during the three months postoperatively compared with group K (*P*=0.05) (Table 5). The incidence of discomfort and radiating pain was higher in group S than in group K. Complications like discomfort, radiating pain at 3 months were present in 13/29 (44.8%) patients in group S and in 05/28 (17.9%) patients in group K. Oral analgesics were administered to two patients in group S and one patient in group K.

## Discussion

In the present study, the NLR and PLR at 2 hours after laparoscopic inguinal hernia surgery and POD1 from preoperative values were increased in both groups. Kim et al.^1^ also observed an increase in NLR until 24 hours after laparoscopic assisted vaginal hysterectomy. An increase in NLR signifies compromised immunity and activation of inflammatory response.^1^ Previous studies have shown that NLR and PLR are reliable markers of inflammation and can predict disease-free survival rates, postoperative morbidity, and mortality.^4,5,6,8,9^ Tzikos et al.^19^ noted that NLP and PLR could be a good predictor for 90-day mortality and length of hospital stay in patients undergoing cardiac surgery. The mean NLRs in normal males and females were 1.63 (0.76) and 1.66 (0.82).^20^ We observed comparable preoperative NLR between 1.4-2.79 in both groups. Preoperative NLR can be used as a predictor of the severity of appendicitis, with more than 8 values for severe acute appendicitis.^21^ Higher preoperative NLR also helps in the diagnosis of requirement for intestinal resection for incarcerated hernia.^5^ An increase in NLR of more than 2.5-5 times from baseline or normal values is considered a poor prognostic indicator in cancer surgeries.^4^

Silva et al.^3^ concluded that >10 NLR on the first postoperative day may be a surrogate marker for increased complications due to significant inflammation after bariatric surgery. The authors did not note NLR before the first postoperative day. In the present study, the NLR increased to 14.7 at 2 hours postoperatively; returned to 7.55 on POD1 in the saline group, whereas in the ketamine group, the NLR remained below 7.61 postoperatively. We observed that the increase in NLR at 2 hours after surgery from baseline was significantly less i.e. 2.53 times in comparison with 4.63 times in the saline group, suggesting suppression of the inflammatory response after surgery with ketamine. Ketamine acts at various levels during the inflammatory process, including in inflammatory cell recruitment, cytokine production, and the regulation of inflammatory mediators. However, this effect was short term, as the POD1 increase in NLR in both groups was comparable.

The mean reference value of the PLR was 132.40 (46.79-218.01).^20^ We observed the basal PLR value in the range of 45.57-78.91. Turkmen et al.^6^ concluded that the PLR is a better inflammatory marker than NLR for predicting inflammation in end-stage renal disease and also showed its positive correlation with NLR, CRP, IL6, and TNF-α. PLR alone was also considered for predicting major surgical complications after pancreatico-duodenostomy, with an optimal cutoff of 145.3 within 30 days.^8^ In the present study, the maximum PLR at two hours postoperatively with low-dose ketamine was 174.79 and with saline was 305.89; which decreased to 121.41 and 169.49 on POD1, respectively. The increase in PLR was significantly less (1.94 time) at 2 hours postoperatively with ketamine in comparison to 2.98 times with saline is due to the anti-inflammatory effect of low-dose ketamine infusion. The comparable PLRs on POD1 in both groups suggest a short-term effect of ketamine infusion. Previous studies have also shown that low-dose ketamine resulted in decreased levels of inflammation markers (IL6) during prolonged open abdominal surgeries and cardiopulmonary bypass with hemodynamic changes.^10, 22^

Surgical procedures are associated with altered homeostasis, leading to the release of stress hormones, pain, and inflammatory reactions. Systemic inflammatory response includes leukocytosis, neutrophilia, lymphopenia, apoptosis of lymphocytes or inhibition of apoptosis of neutrophils.^1^ Neutrophilia represents low-grade to unrestrained cellular inflammation, whereas lymphopenia is indicative of latent immune response. Platelets participate in microcirculation thrombosis at the surgical site, hampering blood supply to the surgical wound and altering the healing process, resulting in decreased platelet counts. We also observed leukocytosis, neutrophilia, lymphopenia, and decreased platelet count postoperatively in both groups until 24 hours after hernia surgery, but neutrophilia and lymphopenia were significantly less with ketamine infusion at 2 hours after surgery compared with saline infusion. Predictors of postoperative infection include significant neutrophilia and lymphopenia after surgery. Our findings suggested that the anti-inflammatory effect of intraoperative ketamine infusion as an increase in NLR and decrease in LC is present in sepsis and bacteraemia.^7^

We selected only laparoscopic inguinal hernia surgery, which had less hemodynamic changes and minimal blood loss, to avoid confounding factors like surgical procedure and duration of surgery, to evaluate the effect of low-dose ketamine on inflammatory markers. Comparable demographic data and comorbidities avoided additional confounding factors in the present study, such as diabetes, hypertension, and asthma, which may lead to change in values of NLR and PLR.^23,24,25,26^ Our anaesthesia technique was also similar in both groups, except for the study drug to eliminate further bias as anaesthetic drugs can modify inflammation and pain during and after surgery.^1, 2, 27^ Domagalska et al.^28^ observed that erector spinae plane block lowers NLR and PLR ratios 12 and 24 hours after spinal surgery. Kim et al.^1^ compared total intravenous anaesthesia with propofol and remifentanil with sevoflurane anaesthesia in laparoscopic assisted vaginal hysterectomy and observed a significant decrease in NLR during the immediate postoperative period and two hours after surgery with TIVA compared with sevoflurane anaesthesia. Similar to our finding, the difference in NLR values was for short term, as NLR values were comparable at 24 hours postoperatively.^1^ The short term effect on NLR may be due to inclusion of minor laparoscopic surgeries.

In the present study, ketamine infusion significantly reduced fentanyl requirement during the intraoperative and postoperative periods. Our results are similar to those of previous studies in which ketamine was administered at a low-dose bolus or bolus followed by intraoperative infusion, resulting in decreased opioid requirement perioperatively.^13, 14^ Pain after laparoscopic inguinal hernia surgery is mainly due to peritoneal stretching caused by gas insufflation and diaphragmatic irritation. Ketamine provides preemptive analgesia by inhibiting central sensitization of pain and inflammation during and after surgery.^29^

Similar to previous findings, we found no difference in hemodynamics, extubation, side effects like emergence delirium, shivering, nystagmus, and PONV, between the low-dose intraoperative ketamine and saline groups.^11, 12, 13, 14^ Increase in time to follow verbal command and awakening was statistically significant in the ketamine group, whereas clinical delay of 2-3 minutes was not significant.

The incidence of chronic pain was significantly less in ketamine group in comparison to the saline group. Chronic post hernia pain syndrome after laparoscopic hernia surgery has somatic and neuropathic components. Somatic pain is caused by damage to the pubic tubercle during the stapling of the mesh prosthesis or deep muscle layers. Neuropathic pain is probably due to primary damage to the ilioinguinal or genitofemoral nerve. Secondary nerve damage can occur due to irritation or compression by an adjacent inflammatory process, such as granuloma.^30^ Ketamine decreases the incidence of chronic pain by acting on NMDA receptors, activating descending inhibitory pathways arising from supraspinal sites, and inhibiting dorsal horn nociceptive neurons. A systematic review and meta-analysis concluded that low-dose ketamine infusion both intraoperatively and postoperatively decreased the incidence of chronic pain.^17^ The timing and dosage of ketamine bolus and infusion in different studies were highly variable. We administered 0.5 mg kg^-1^ bolus ketamine at induction followed by 0.2 mg kg^-1^ h^-1^ infusion till the end of surgery. In contrast, Kwok et al.^11^ did not find any difference in the incidence of chronic pain  one month after laparoscopic gynecological surgeries with the pre-incision 0.15 mg kg^-1^ ketamine bolus.

### Study Limitations

The limitations of the present study were the inclusion of laparoscopic surgery with less surgical trauma and the inability to assess the correlation of NLR and PLR with chronic pain due to the small sample size.

## Conclusion

This study suggested that low-dose intravenous ketamine bolus followed by infusion reduced the inflammatory response, as observed by a significant decrease in the NLR and PLR values at 2 hours postoperatively from baseline after laparoscopic inguinal hernia surgery. Ketamine bolus infusion reduces perioperative and chronic pain at 3 months after laparoscopic hernia surgery, with a minimal delay in recovery without any side effects.

## Ethics

**Ethics Committee Approval:** This prospective, randomized, double-blind study was approved by the Institutional Ethics Committee for Post Graduate Research All India Institute of Medical Sciences, Ansari Nagal, New Delhi for this study (approval no.: IECPG-268/28.06.2018, date: 26.07.2018) and registered with the Clinical Trials Registry, India, (CTRI/2018/08/015320).

**Informed Consent: **Written informed consent was obtained from the patients before recruitment.

## Figures and Tables

**Table 1. Demographic Data, Comorbidities of Patients, Type of Inguinal Hernia and Surgical Procedure:** 

			**Group K, n = 30 (%)**	**Group S, n = 30**	***P *value**
**Age, years (Mean ± SD, 95% CI)**			38.9±13.1 (34.1-43.9)	38.2±13.6 (33.2-43.4)	0.84
**Gender**		**Male**	29 (96.7)	30 (100)	0.31
		**Female**	1 (3.3)	0 (0)	
**Weight, kg (Mean ± SD, 95% CI)**			62.3±8.7 (59.1-65.6)	65.3±10.6 (61.3-69.2)	0.25
**Comorbidities**		**None**	22 (73.3)	27 (90)	0.09
		**Hypertension**	7 (23.3)	1 (3.3)	
		**Diabetes**	1 (3.3)	0 (0)	
		**Asthma**	0 (0)	1 (3.3)	
		**Hypertension and diabetes**	0 (0)	1 (3.3)	
**Type of inguinal hernia**	**Direct**	**Bilateral**	2 (6.7)	2 (6.7)	0.9
		**Right-sided**	3 (10)	2 (6.7)	
		**Left-sided**	1 (3.3)	0 (0)	
	**Indirect**	**Bilateral**	6 (20)	8 (26.7)	
		**Right-sided**	12 (40)	13 (43.3)	
		**Left sided**	6 (20)	5 (16.7)	
**Type of surgery**		**TAPP**	16 (53.3)	12 (40)	0.3
		**TEP**	14 (46.7)	18 (60)	
**Duration of surgery, minutes (Mean ± SD, 95% CI)**			60.5±18.6 (55.8-68.5)	62.2±17 (53.5-67.5)	0.71

n, number of patients; SD, standard deviation; CI, confidence interval.

**Table 2. Perioperative Fentanyl Requirement and Recovery After Anaesthesia:** 

		**Group K, n = 30** **Mean±SD (95% CI)**	**Group S, n = 30** **Mean±SD (95% CI)**	***P *value**
**Fentanyl (µg)**	**At induction**	126.2±17.9 (119.52-132.88)	127.8±18.1 (121.08-134.59)	0.72
	**Intraoperative period**	7±15.2 (1.35-12.65)	26.6±28.3 (16.07-37.2)	0.001*
	**Postoperative period**	40.3±13.7 (30.5-50.02)	57.7±24.4 (45.6-69.8)	0.047*
**Time taken for extubation (minutes)**		9.4±4.3 (7.8-11)	8.6±3.6 (7.2-9.95)	0.42
**Time taken to follow verbal commands (minutes)**		13.7±5.9 (11.5-15.9)	10.3±4.0 (8.8-11.8)	0.01*
**Time taken to reach MAS >9 (minutes)**		18.6±5.8 (16.5-20.8)	16±4.2 (14.4-17.6)	0.05*

*Statistically significant.

mg, milligram; µg, microgram; MAS, modified Aldrete score; n, number of patients; SD, standard deviation; CI, confidence interval.

**Table 3. Total Lymphocyte Count, Neutrophil Count, Platelet Count, Lymphocyte Count, Neutrophil Lymphocyte Ratio and Platelet Lymphocyte Ratio at Various Time Intervals:** 

		**Group K, n = 30** **Median (IQR)**	**Group S, n = 30** **Median (IQR)**	***P* value**
**TLC (Number of cells x 10^6^/liter)**	T0	6815 (5650-7590)	6790 (5572-7287)	0.65
	T1	9700 (7520-14635)	11450 (9375-14687)	0.32
	T2	7455 (5725-9445)	8400 (1057-11040)	0.13
**NC (% of TLC)**	T0	59.5 (48.25-63.5)	60 (53-65.75)	0.21
	T1	77 (67.5-80)	84 (80-88.75)	0.0005*
	T2	72.5 (68-75.75)	75 (69.3-81.5)	0.28
**LC (% of TLC)**	T0	31.5 (25-37.95)	29 (24-34.25)	0.51
	T1	14.5 (10.25-21.5)	11 (6.25-14.5)	0.01*
	T2	18.5 (15-21.98)	18 (11-20.75)	0.49
**PC (number of cells **× **10^11^/liter)**	T0	1.85 (1.36-2.05)	1.71 (1.50-1.98)	0.80
	T1	1.64 (1.23-2.12)	1.66 (1.42-2.18)	0.41
	T2	1.58 (1.34-2.06)	1.81 (1.6-2)	0.21
**NLR (ANC/ALC **× **100)**	T0	1.85 (1.4-2.61)	2.07 (1.72-2.79)	0.47
	T1	5.45 (2.89-7.61)	7.91 (5.74-14.7)	0.007*
	T2	4.14 (2.93-5.05)	4.21 (3.39-7.55)	0.34
	Number of times rise in NLR at T1 from T0	2.53 (1.73-4.56)	4.63 (2.72-6.43)	0.02*
	Number of times rise in NLR at T2 from T0	2.41 (1.66-3.43)	2.23 (1.66-4.37)	0.81
**PLR (APC/ALC **× **100)**	T0	55.2 (52.03-78.91)	58.9 (45.57-76)	0.99
	T1	124.55 (66.45-174.79)	157.78 (127.42-305.89)	0.03*
	T2	97.77 (66.67 -121.41)	126.14 (76.31-169.49)	0.19
	Number of times rise in NLR at T1 from T0	1.94 (1.38-3.21)	2.98 (2.11-4.4)	0.02*
	Number of times rise in NLR at T2 from T0	1.73 (1.37-2.03)	2.01 (1.27-2.95)	0.41

*Statistically significant.

IQR, interquartile range; TLC, total leucocyte count; NC, neutrophil count; ANC, absolute neutrophil count (%NC/deciliter); PC, platelet count; APC, absolute platelet count (PC/deciliter); LC, lymphocyte count; ALC, absolute lymphocyte count (%LC/deciliter); n, number of patients; T0, preoperative; T1, two hours postoperatively; T2, postoperative day one.

**Table 4. Postoperative Verbal Analogue Score:** 

	**VAS (0-3)**		**VAS (>3)**		***P *value**
**Time (minutes)**	**Group K n = 30 (%)**	**Group S n = 30 (%)**	**Group K n = 30 (%)**	**Group S n = 30 (%)**	
**0**	27 (90)	17 (56.7)	3 (10)	13 (43.3)	0.007*
**10**	25 (83.3)	26 (56.7)	5 (16.7)	4 (13.3)	0.99
**30**	28 (93.3)	28 (93.3)	2 (6.7)	2 (6.7)	0.99
**60**	24 (80)	24 (80)	6 (20)	6 (20)	0.99
**120**	28 (93.3)	25 (83.3)	2 (6.7)	5 (16.7)	0.42

*Statistically significant.

n, number of patients; VAS, verbal analogue score.

**Table 5. Incidence, Symptoms and Requirement of Medication for Chronic Pain:** 

		**Group K n = 28 (%)**	**Group S n = 29 (%)**	***P *value**
**VAS after three months**	**0**	20 (71.4)	16 (55.2)	0.05*
	**1**	1 (3.6)	0 (0)	
	**2**	1 (3.6)	1 (3.4)	
	**3**	2 (7.1)	11 (37.9)	
	**4**	1 (3.6)	4 (13.8)	
**Three months postoperatively**	**No complaints**	23 (82.1)	16 (55.2)	0.09
	**Discomfort**	1 (3.6)	2 (6.9)	
	**Radiating pain**	4 (14.3)	11 (37.9)	
	**Not on medication**	27 (96.4)	27 (93.1)	0.57
	**Paracetamol for pain**	1 (3.6)	2 (6.9)	

*Statistically significant.

n, number of patients; VAS, verbal analogue score.
